# Supplementary material for: Comparison of Four Systems for SARS-CoV-2 Antibody at Three Time Points after SARS-CoV-2 Vaccination
Source: Diagnostics (Basel). 2022 May 29;12(6):1349. doi: 10.3390/diagnostics12061349 (PMC9222035; doi:10.3390/diagnostics12061349)
Supplement: Supplementary file 1 [file diagnostics-12-01349-s001.zip › diagnostics-1753623-supplementary.pdf]

## Supplementary Materials

**Table S1**. The sensitivity and specificity of each test system at manufacturer-claimed cutoff and estimated optimal cutoff by the alteration of cPASS cutoff.

| | Cutoff | 50% inhibition | | 70% inhibition | | 90% inhibition | |
|---|---|---|---|---|---|---|---|
| | | Sn (95% CI*) | Sp (95% CI) | Sn (95% CI) | Sp (95% CI) | Sn (95% CI) | Sp (95% CI) |
| sCOVG | Claimed | 99.81 (98.9–100.0) | 28.39 (22.7–34.6) | 100.0 (99.0–100.0) | 17.44 (13.8–21.6) | 100.0 (98.2–100.0) | 12.45 (9.8–15.5) |
| | Optimal | 90.25 (87.4–92.7) | 83.90 (78.6–88.3) | 89.42 (85.8–92.4) | 78.21 (73.8–82.2) | 96.06 (92.4–98.3) | 76.74 (73.0–80.2) |
| CoV-2 IgG II | Claimed | 100.0 (99.3–100.0) | 8.05 (4.9–12.3) | 100.0 (99.0–100.0) | 4.87 (3.0–7.5) | 100.0 (98.2–100.0) | 3.48 (2.1–5.4) |
| | Optimal | 91.26 (88.5–93.6) | 84.75 (79.5–89.1) | 94.74 (91.9–96.8) | 77.44 (73.0–81.5) | 92.68 (88.2–95.8) | 83.88 (80.5–86.9) |
| Immuno-On IgG | Claimed | 97.09 (95.2–98.4) | 26.69 (21.2–32.8) | 97.78 (95.7–99.0) | 17.95 (14.3–22.1) | 98.54 (95.8–99.7) | 28.94 (25.2–32.9) |
| | Optimal | 82.14 (78.5–85.3) | 78.39 (72.6–83.5) | 80.89 (76.4–84.8) | 80.77 (76.5–84.6) | 84.39 (78.7–89.1) | 82.60 (79.2–85.7) |

The estimated cutoffs for each system that correspond to altered neutralization cutoff; 50%, 70%, and 90% inhibition were used as "optimal" cutoff in this analysis. * If there is no overlap in the 95% CIs between parameters, it can be interpreted as having a statistically significant difference at the significance level of 0.05 (5%). **Abbreviations**: Sn, sensitivity; Sp, specificity; CI, confidence interval; cPASS, GenScript cPASS SARS-CoV-2 neutralization antibody detection kit; sCOVG, Siemens SARS-CoV-2 IgG; CoV-2 IgG II, Abbott SARS-CoV-2 IgG II Quant; Immuno-On IgG, Osang Immuno-On™ COVID-19 IgG test.

| A | | | |
|---|---|---|---|
| Immuno-On IgG | 0.748 (0.716-0.779) | 0.562 (0.526-0.598) | 0.370 (0.336-0.406) |
| CoV-2 IgG II | 0.710 (0.676-0.742) | 0.505 (0.468-0.541) | 0.297 (0.264-0.331) |
| sCOVG | 0.772 (0.741-0.802) | 0.570 (0.534-0.606) | 0.362 (0.328-0.398) |
| | 50% inhibition | 70% inhibition | 90% inhibition |

| B | | | |
|---|---|---|---|
| Immuno-On IgG | 0.808 (0.778-0.836) | 0.807 (0.777-0.835) | 0.830 (0.801-0.856) |
| CoV-2 IgG II | 0.891 (0.866-0.912) | 0.856 (0.829-0.881) | 0.862 (0.835-0.885) |
| sCOVG | 0.880 (0.855-0.903) | 0.834 (0.805-0.860) | 0.819 (0.790-0.846) |
| | 50% inhibition | 70% inhibition | 90% inhibition |

**Figure S1.** Concordance and 95% CI of qualitative decision of each test system with cPASS following the change in % inhibition cutoff for neutralization, (A) when the manufacturer-claimed cutoff for each test was applied or (B) the estimated cutoff for each test corresponding to change of neutralization cutoff was applied. If there is no overlap of 95% CIs, it can be interpreted as having a statistically significant difference at the significance level of 0.05.

**Acknowledgement**

Siemens Healthcare Diagnostics Inc., Abbott Laboratories, and Osang Healthcare Inc. showed their support through the provision of anti-SARS-CoV-2 IgG test kits. Siemens, Abbott, and Osang did not play any role in the study design or data analysis.

**Author contributions**

All authors have accepted responsibility for the entire content of this manuscript and approved its submission. J. D. Seo collected and analyzed the data and wrote the initial draft. H.-W. Moon designed and supervised the study and finalized the draft. M. Nam collected and analyzed the data. T. H. Lee, Y.-S. Ahn, S.-H. Shin, and H.Y. Han collected the samples and performed the tests.

**Institutional Review Board Statement**

This study was conducted after obtaining approval from the Institutional Review Board of our institution. (IRB No. KUMC 2021-05-033)

**Informed Consent Statement**

All subjects were ≥18 years of age at enrollment and written informed consent was obtained.

**Data Availability Statement**

A data set of serological responses of 930 samples was deposited at https://dataverse.harvard.edu/ (https://doi.org/10.7910/DVN/CMRD77).

**Conflict of Interest**

The authors declare no conflicts of interest.
